# Quantitative and Microstructural Changes of the Blood-Nerve Barrier in Peripheral Neuropathy

**DOI:** 10.3389/fnins.2018.00936

**Published:** 2018-12-18

**Authors:** Ann Kristin Reinhold, Joachim Schwabe, Thomas J. Lux, Ellaine Salvador, Heike L. Rittner

**Affiliations:** Department of Anaesthesiology, University Hospitals Würzburg, Wüerzburg, Germany

**Keywords:** neuropathic pain, chronic constriction injury, blood-nerve barrier, tight junction protein, claudin-1, ZO-1

## Abstract

Peripheral neuropathy is accompanied by changes in the neuronal environment. The blood-nerve barrier (BNB) is crucial in protecting the neural homeostasis: Tight junctions (TJ) seal paracellular spaces and thus prevent external stimuli from entering. In different models of neuropathic pain, the BNB is impaired, thus contributing to local damage, immune cell invasion and, ultimately, the development of neuropathy with its symptoms. In this study, we examined changes in expression and microstructural localization of two key tight junction proteins (TJP), claudin-1 and the cytoplasmic anchoring ZO-1, in the sciatic nerve of mice subjected to chronic constriction injury (CCI). Via qPCR and analysis of fluorescence immunohistochemistry, a marked downregulation of mRNA as well as decreased fluorescence intensity were observed in the nerve for both proteins. Moreover, a distinct zig-zag structure for both proteins located at cell-cell contacts, indicative of the localization of TJs, was observed in the perineurial compartment of sham-operated animals. This microstructural location in cell-cell-contacts was lost in neuropathy as semiquantified via computational analysis, based on a novel algorithm. In summary, we provide evidence that peripheral neuropathy is not only associated with decrease in relevant TJPs but also exhibits alterations in TJP arrangement and loss in barrier tightness, presumably due to internalization. Specifically, semiquantification of TJP in cell-cell-contacts of microcompartments could be used in the future for routine clinical samples of patients with neuropathy.

## Introduction

Neuropathic pain is defined as pain due to a lesion or disease of the somatosensory nervous system. Previous studies have shown that in several neuropathic conditions, one critical factor is a deranged homeostasis in the neural milieu, e.g., through invasion of immune cells and local production or influx of inflammatory mediators like cytokines (Sommer and Schafers, 1998; Gaudet et al., 2011). Recently, Uceyler et al. (2016) showed that endoneurial edema in the sural nerve is indicative of recent onset in patients with both inflammatory and non-inflammatory neuropathy. Not only do small changes in ion concentrations (e.g., potassium, sodium, and calcium) or pH profoundly disturb neuronal conduction; moreover, blood-borne factors such as cytokines or microbial agents may directly damage the nerve (Gaudet et al., 2011). As a consequence, the peripheral nerve is protected by the blood-nerve barrier (BNB).

The BNB emanates largely from two compartments, the endothelium of endoneurial vessels, and the perineurium, surrounding a nerve fascicle. Blood-borne damaging mediators can either reach the nerve via permeable endoneurial vessels or by leakage from epineurial vessel, via the perineurium. Adherens proteins, e.g., junctional adhesion molecule-C (JAM-C), are responsible for the linkage of cells. Gap junctions and their proteins, connexins, connect the cytoplasm of two cells, allowing selected molecules to directly pass between cells. Tight junctions (TJ) are built by selected tight junction proteins (TJP) at cell-cell contacts. They seal the intercellular space through formation of a paracellular barrier, thereby controlling influx of molecules. Both barriers, the perineurium and the endoneurial vessels, are assembled by a distinctive set of proteins to maintain homeostasis (Reinhold and Rittner, 2017). In this study, we focus on the perineurium as one central and well-characterized part of the BNB. Claudin-1 is a crucial TJP in maintaining the BNB – specifically the perineurium. This prominent member of the claudin family is a 22 kDa protein, present in various tissues (Furuse et al., 1998). For example, it is found in the gut, the kidney and the skin. Claudin-1 knockout mice die from dehydration due to its loss in the epidermis (Furuse et al., 2002). Decreased claudin-1 expression dose-dependently alters the severity of atopic dermatitis and skin barrier function (Tokumasu et al., 2016). Claudin-1 is expressed in the PNS (and the CNS, Liebner et al., 2000; Pfeiffer et al., 2011) and plays a pivotal role in maintaining the BNB (Hackel et al., 2012; Rittner et al., 2012; Zwanziger et al., 2012; Sauer et al., 2014; Dabrowski et al., 2015). In the peripheral nerve, it is found mainly in the perineurium and, to lesser degree, in endoneurial vessels (Pummi et al., 2004). Claudin-1 binds to the PDZ domain of the cellular actin cytoskeleton via zona-occludens 1 (ZO1) protein, a cytoplasmatic multi-domain scaffolding protein (Itoh et al., 1999). ZO-1 deficiency in mice is associated with a lethal phenotype (Katsuno et al., 2008). In several neurological disorders, a downregulation of TJP is associated with TJ disruption and, ultimately, barrier breakdown. Neuroinflammatory diseases such as Guillain-Barré syndrome (GBS) and chronic inflammatory demyelinating polyneuropathy (CIDP) present with TJP downregulation and accompanying BNB leakage (Kanda, 2013; Ubogu, 2015).

Previous studies have shown leakage of the BNB including a reduction in TJP expression in different neuropathies like nerve crush, chronic constriction injury (CCI) and partial sciatic nerve ligation (Hirakawa et al., 2003; Lim et al., 2014; Moreau et al., 2016). Here, we used CCI, the established rodent model of traumatic peripheral mononeuropathy (Bennett and Xie, 1988). CCI consists of a loose ligation of the sciatic nerve, leading to partial damage to the nerve (Reinhold et al., 2015) and results in mechanical and thermal hypersensitivity after up to 7 days depending on the species (Sauer et al., 2017). In rats, BNB leakage is observed as early as 6 h after surgery, downregulation of TJP such as claudin-5 and occludin can be detected even earlier (Moreau et al., 2016). Especially claudin-5 is a typical TJP of endoneurial vessels and not present in the perineurium. In their study in CCI, BNB opening starts distinctly before the onset of neuropathic symptoms. However, claudin-5 mRNA expression is at its minimum after 7 days, i.e., concordant with the phenotype (Moreau et al., 2016).

Most research on the BNB to date has focused on overall quantitative changes of TJP mRNA and protein expression after neuropathy disregarding the compartmental distribution of the TJP. To estimate the relevance of such observations, however, the accompanying structural changes and exact localization of such quantitative changes need to be taken in account.

In this study, we measured alterations in TJP, both quantitatively and microstructurally in cell-cell-contacts. The key TJPs claudin-1 and ZO-1 were quantified in the nerve using qPCR. Semiquantification specific to different compartments of the BNB was performed on immunohistochemical stainings of murine nerve tissue after CCI. To this end, an image-based algorithm was developed. Co-staining of both proteins allowed for assessment of their respective localization. Structural changes were assessed using a computer-based analysis of the images and compared to scorings by blinded expert scientists.

## Materials and Methods

### Animals

Sixteen adult 9–12 weeks old male C57BL/6 mice (Janvier, Saint Berthevin Cedex, France) were kept in cages of four in a 12 h light cycle, with water and food *ad libitum*. Animal protocols were approved by the provincial government of Würzburg (Regierung von Unterfranken, Germany) and are in accordance with the guidelines of the International Association for the Study of Pain (IASP, 2018).

### Chronic Constriction Injury

Mice underwent surgery under deep isoflurane anesthesia (1.8 Vol%, fiO2 1.0). Absence of paw withdrawal indicated adequate surgical anesthesia. For CCI, the sciatic nerve at the right midthigh was exposed after skin incision and blunt preparation through the muscles, and three loose silk ligatures (Perma Silk 6.0, Ethicon Inc., Somerville, NJ, United States) with about 1-mm spacing were loosely tightened around the sciatic nerve (Reinhold et al., 2015). Great care was taken to avoid harming the nerve with surgical tools. The wound was closed with sutures (Prolene 5.0, Ethicon Inc., Somerville, NJ, United States). In sham-operated mice, the sciatic nerve was briefly exposed without introducing or performing ligatures. The wound was closed with sutures.

### qPCR

mRNA quantification was carried out using qPCR of the ipsilateral sciatic nerve 7 days after CCI or sham surgery. GAPDH served as a reference gene. Assays were used as predesigned by the manufacturer (claudin-1: Mm00516701_m1, ZO-1: Mm00493699_m1, GAPDH: Mm99999915_g1, all Invitrogen, Thermo Fisher, Waltham, MA, United States). For reverse transcription and qPCR, Invitrogen TaqMan^®^ MicroRNA Reverse Transcription Kit and Applied Biosystems^TM^ TaqMan^®^ Fast Universal PCR Master Mix (2X), no AmpErase UNG (Thermo Fisher, Waltham, MA, United States) were used following the manufacturer’s miRNA standard protocol. The qRT-PCR was conducted with the StepOnePlus real-time PCR system (Thermo Fisher, Waltham, MA, United States). *C*t values were calculated for each replicate. Expression was analyzed using the ΔCt method (2^∧^- Δ*C*t = 2^∧^-(*C*t(CCI)-*C*t(Sham)).

### Immunofluorescence and Microscopy

Seven days after surgery, the mice were euthanized. The sciatic nerve was harvested, snap-frozen in liquid nitrogen, embedded in Tissue Tek O.C.T. Compound (Sakura Finetek Europe B.V., AV Alphen aan den Rijn, Netherlands) and stored at -20°C. Using a cryostat, 10 μm-sections were cut at -20°C distal and proximal to the ligatures (Leica Biosystems CM3050 S Research Cryostat, Leica Biosystems Nussloch GmbH, Nussloch, Germany).

For immunofluorescence labeling, samples were fixed in ethanol (Sigma-Aldrich, St. Louis, MO, United States), permeabilized with 0.5% Triton X-100 (Sigma-Aldrich, St. Louis, MO, United States) in PBS and blocked with 10% donkey serum in PBS. Afterward the sections were immediately incubated with the following primary antibodies (in 10% donkey serum in PBS) overnight at 4°C: goat ZO-1 antibody (1:150, Biorbyt San Francisco, CA, United States, orb153344) and/or rabbit anti-claudin-1 (1:100, Thermo Fisher, Rockford, IL, United States, 51-9000). For co-stainings, sections were incubated with both primary antibodies against claudin-1 and ZO-1. After washing in PBS, nerve samples were incubated with secondary antibodies (in PBS): For claudin-1, Alexa Fluor 555 donkey anti-rabbit IgG (H+L) (1:1.000, Life Technologies, Invitrogen, Molecular Probes Inc., Eugene OR, United States, A31572) and for ZO-1, Alexa Fluor 488 donkey anti-goat IgG (H+L) (1:1.000, Life Technologies, Invitrogen, Molecular Probes Inc., Eugene OR, United States, A11055) was employed. Nuclei were stained with 4′,6-diamidino-2-phenylindole (DAPI – 2 mg/ml in distilled water, Roche Diagnostics, Mannheim, Deutschland) in PBS (1:2.000) for 10 min. After washing, the sections were mounted with Vectashield Antifade Mounting Medium (Vector Laboratories, Burlingame, CA, United States). Stainings were visualized by immunofluorescence microscopy (Biorevo BZ-9000-E, Keyence, Osaka, Japan) with the same settings for each antibody. Z-stacks (1 μm) were saved in RGB 8-bit Tagged Image File Format (TIFF) pictures (see below).

### Computational Analysis

**FIGURE 1:**
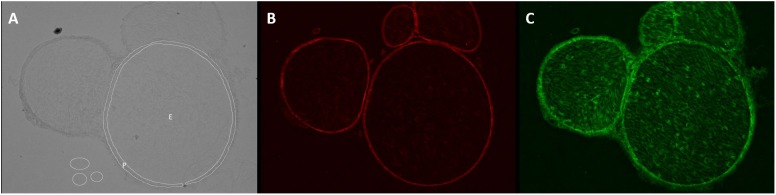
Determination of endo- and perineurium. Brightfield and color channels of a sample sciatic nerve preparation. **(A)** The white circles are plotted in the brightfield channel to denote ROI as endoneurium (E) and perineurium (P). They are then projected on the color channels. **(B)** Claudin-1 fluorescence is strictly perineurial while **(C)** ZO-1 is located both endo- and perineurially.

Unchanged RGB-8-bit images were processed with Fiji/ImageJ (versions 1.51d-j and 1.52e, Open Source). For *semiquantification of the total immunoreactivity*, endoneurium, and perineurium were manually defined in captured brightfield images, saved as *region of interest* (ROI) and transferred to the immunofluorescence images (Figure 1). Immunofluorescence intensity was measured in images with same settings and magnification in three adjacent z-stacks (step size 1 μm). Claudin-1 intensity was assessed in the perineurium, ZO-1 intensity in both perineurium and endoneurium. Intensity was determined with the integrated *measure* function in Fiji/ImageJ [determined intensity (IntD) = “selected area” ^∗^ “mean color intensity”]. To correct the intensity for noise (corrected immunofluorescence intensity, IntC), the background signal was registered in three areas and the given mean subtracted from the determined intensity [IntC = IntD – (“selected area” ^∗^ “mean of the color intensity of 3 background areas”)]. By referring to the initially selected area, a normalized immunofluorescence intensity (NIFI) was determined (NIFI = IntC / selected area). The mean NIFI from up to three fascicles of each animal was used to quantify and compare stainings. NIFI means of the three groups (Sham, CCI proximal, CCI distal) were compared.

**FIGURE 2:**
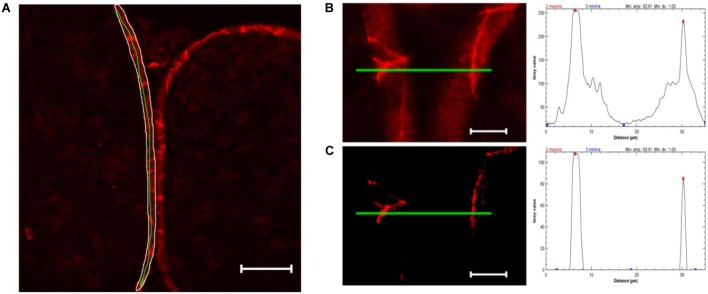
Computational peak analysis. **(A)** Sample of a sham nerve after claudin-1 immunohistochemistry (scale bar 50 μm). Marked is a representative perineurial ROI (white), to define the gray value mean and standard deviation, and a green line used for peak analysis. **(B)** Magnified selection of **(A)** and putative peak analysis (green line). **(C)** Selection of **(B)** after substracting 1.5 ^∗^ mean gray value (determined in perineurium ROI) and applying Gaussian blur (*r* = 2 pixels) and putative peak analysis. Peak analysis shows drastically reduced background signal not affecting strong peaks.

For *semiquantification of TJPs in cell-cell contacts*, only images of claudin-1 fluorescence were used. The perineurium in each sample was manually defined as ROI. The mean value and standard deviation of all pixels in the perineurium were calculated using ImageJ’s integrated *measure* function. Unspecific signals were eliminated by subtracting mean gray value ^∗^1.5 using the integrated *Gaussian blur* function (radius = 2 pixels). For each sample, peaks ( = cell-cell contacts) were analyzed in five manually drawn lines over the whole perineurium length. To determine peaks, the *Find Peak* function was used (Fiji’s collection of “broadly applicable routines” (Ferreira et al., 2017), settings: minimal peak amplitude = 1.5^∗^standard deviation, minimal peak distance = 1 μm, minimal value of a maximum = 40). Samples with a mean gray value of ≥143.33 were discarded as they are unable to produce enough contrast to detect peaks (*n* = 1). The mean peak value of five lines was used for further analysis (Figure 2).

### Structural Evaluation

For structural evaluation, two blinded external reviewers with expertise in the field rated the stainings of the perineurium in fluorescent images (*n* = 7) using a questionnaire. Four categories (defined structure/cell-cell contacts, visual contrast, homogeneity of the immunoreactivity and staining quality) were rated on a scale from 1 (none) to 4 (strong/high). Omission of an answer was optional. For training, reviewers were provided with examples for each group. For statistical analysis, experts were analyzed independently.

### Statistical Analysis

For statistical analysis, SigmaPlot (Version 14.01.142, Systat Software GmbH, Erkrath, Germany), RStudio (Version 1.1447, Open Source) and Microsoft Excel 2016 (Version 16.11, Microsoft, Redmond, WA, United States) were used. For *qPCR*, statistical significance was tested via paired *t*-test. To compare groups for *total immunoreactivity*, Friedman ANOVA on ranks was performed, followed by Holm-Sidak *post hoc*-test. For *peak analysis*, Kruskal Wallis test was performed, followed by pairwise Dunn test (one-sided). To analyze *raters’ evaluation*, Friedman RM ANOVA on ranks was used, followed by Holm-Sidak *post hoc* test. Data are depicted as mean +/- SEM. Significance was considered as *p* < 0.05.

## Results

### CCI Induces Structural Changes and Invasion of Nucleated Cells in the Nerve

**FIGURE 3:**
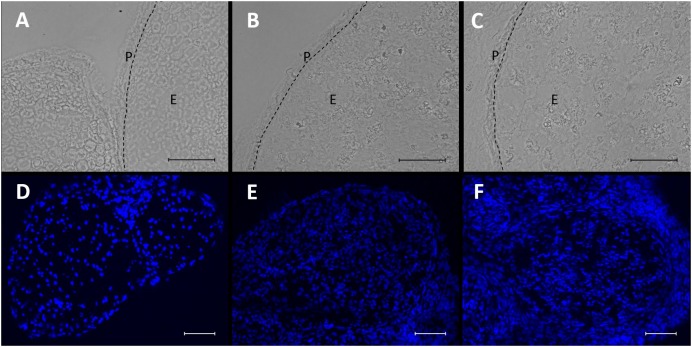
Microstructural changes and invasion of nucleated cells in the nerve after CCI. **(A–C)** Compared to sham control, neural structure is loosened 7 days after CCI both proximal **(B)** and distal **(C)** to the lesion. Scale bar = 50 μm. P, perineurium; E, endoneurium. **(D–F)** Nucleated cells accumulate in the nerve after CCI **(D)** sham, **(E)** CCI, proximal to lesion, **(F)** CCI, distal to lesion. Scale bar = 100 μm.

In brightfield microscopy, sections of the sciatic nerve exhibited a loosening of neural tissue 1 week after CCI (Figures 3A–C). The nerve appeared less compact, and rather teared apart. Axons and myelin sheaths were difficult to distinguish. This was observed in both locations, distal and proximal to the CCI lesion. In addition, CCI led to an accumulation of nucleated cells both around but also within the nerve, as shown via DAPI staining (Figures 3D–F).

### Decreased Claudin-1 mRNA Expression and Immunoreactivity in Cell-Cell Contacts After CCI

**FIGURE 4:**
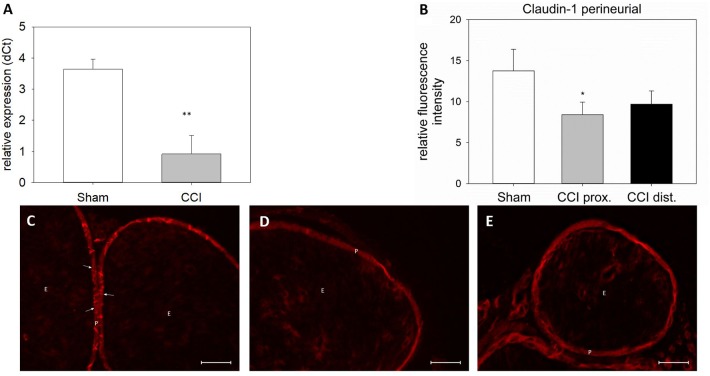
Downregulation and redistribution of claudin-1 in the perineurium. **(A)** Downregulation of claudin-1 mRNA 7 days after CCI (*n* = 8, ^∗∗^*p* < 0.01). **(B)** Decrease in perineurial claudin-1 INFI after CCI (*n* = 7 per group, ^∗^*p* < 0.05). **(C–E)** Structural changes in perineurial claudin-1 after CCI (**C** sham, **D** proximal to lesion, **E** distal to lesion). E, endoneurium; P, perineurium; arrows point to zig-zag structure of cell-cell-contacts in sham controls. Scale bar 50 μm.

**FIGURE 5:**
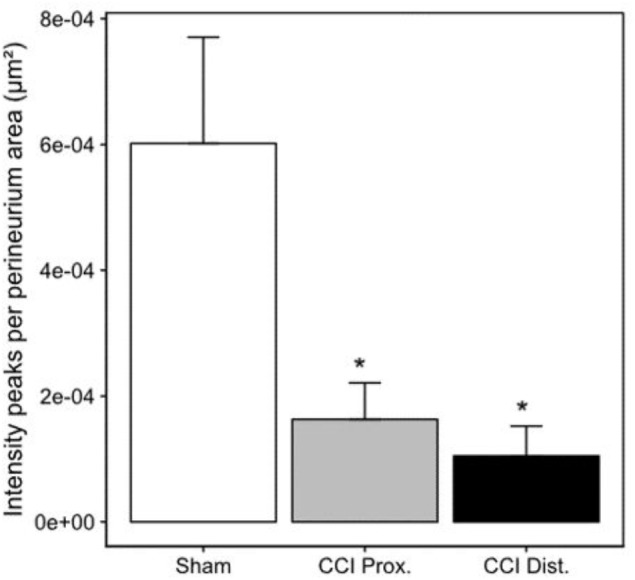
Decrease in perineurial claudin-1 fluorescence peaks. Decrease of mean claudin-1 fluorescence peaks after CCI implies a less stringent expression pattern in the perineurium (*n* = 7 per group, ^∗^*p* < 0.05). Error bars indicate SEM.

**FIGURE 6:**
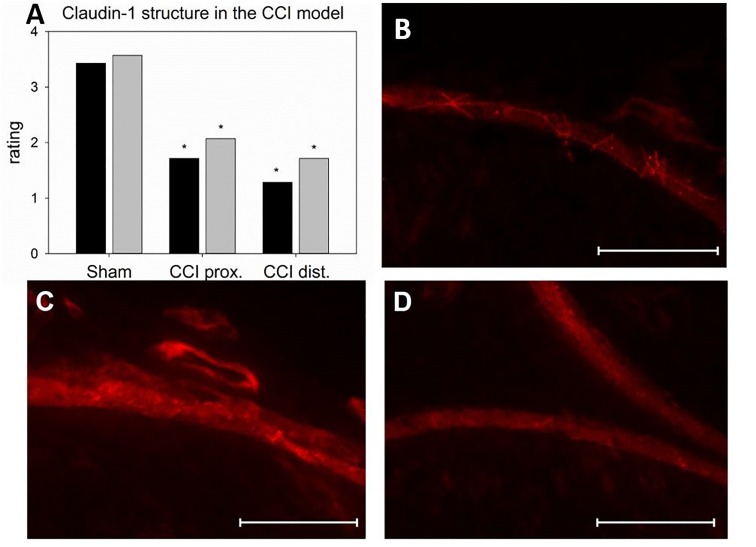
Alterations in the localization of perineurial claudin-1 immunoreactivity. **(A)** Rating of two blinded reviewers (gray, reviewer 1; black, reviewer 2) observing compromised structures after CCI (*n* = 7, ^∗^*p* < 0.05). **(B – D)** example of immunofluorescent sections to be rated (**B** sham, **C** proximal to lesion, **D** distal to lesion). Scale bar 50 μm.

Seven days after CCI, claudin-1 mRNA was downregulated in the sciatic nerve by 74% (*p* < 0.01, Figure 4A). This was in line with NIFI quantification in the perineurium, the main location of claudin-1. (Figure 4B). Proximal to the lesion, NIFI was reduced by 39% (*p* = 0.025). Staining of the distal nerve showed a decrease by 30%, yet not to a significant degree. Moreover, in sham nerves, claudin-1 immunoreactivity in the perineurium was arranged in a sharp zig-zag pattern consistent with cell-cell contacts between perineurial cells (Figure 4C). After CCI, claudin-1 immunoreactivity was notably less clearly localized, hardy observed in expected cell-cell-contacts (Figures 4D,E). Algorithm-based analysis of fluorescence peaks as surrogates for cell-cell-contacts confirmed this observation: Compared to sham, the number of peaks in claudin-1 fluorescence were reduced in CCI, both proximal (by 73%, *p* = 0.013) and distal (by 82%; *p* < 0.05, Figure 5). These findings were in line with ratings by two external expert reviewers blinded to the condition. Both described a significant decrease in two categories, contrast and structure (cell-cell contacts), but not in homogeneity or staining quality (*p* < 0.05, Figure 6). When comparing both methods for semiquantification of the immunoreactivity in the perineurium itself, the effect was more pronounced in the algorithm-based quantification approach. This is very well in line with the observed downregulation of claudin-1 mRNA in the whole nerve.

### Altered Microstructural ZO-1 Immunoreactivity Distribution and Decrease of ZO-1 mRNA After CCI

**FIGURE 7:**
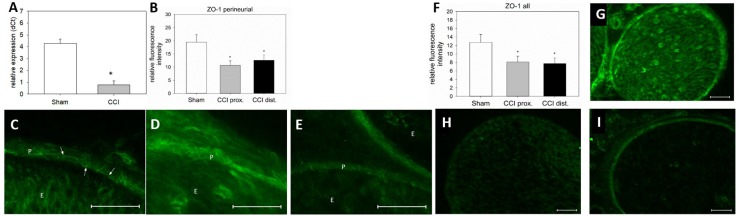
Downregulation and redistribution of ZO-1. **(A)** Downregulation of ZO-1 mRNA 7 days after CCI (*n* = 8, ^∗^*p* < 0.05). **(B)** Decrease in ZO-1 NIFI in the perineurium after CCI (*n* = 7 per group, ^∗^*p* < 0.05). **(C–E)** Quantitative and structural changes in perineurial ZO-1 after CCI (**C** sham, **D** proximal to lesion, **E** distal to lesion). **(F)** Decrease in overall NIFI after CCI (*n* = 7, ^∗^*p* < 0.05), **(G – I)** Quantitative and structural changes in overall ZO-1 after CCI (**G** sham, **H** proximal to lesion, **I** distal to lesion). E, endoneurium; P, perineurium; arrows point to zig-zag structure in sham controls. Scale bar 50 μm.

Seven days after CCI, ZO-1 mRNA was downregulated by 82% in the sciatic nerve as compared to sham-operated animals (*p* < 0.01, Figure 7A). For a more precise evaluation, fluorescence intensity was quantified in two different compartments, the perineurium and the whole nerve. In the perineurium, NIFI was decreased by ca. 40%, compared to sham control. Notably, this was true for both nerve sections, proximal (45% decrease, *p* < 0.01) and distal (35%) to the lesion (*p* = 0.019, Figure 7B). The same was observed in the entire nerve fascicle: proximal to the lesion, NIFI was downregulated by 36%, distal by 39% (*p* = 0.04, Figure 7F). When comparing the arrangement of perineurial ZO-1 staining, the same fine and clear distinguishable zig-zag line indicating cell-cell-contacts was observed in nerve sections from sham-operated animals (Figure 7C, arrows). After CCI, this distribution was markedly less well-defined and, at some point, hard to identify at all (Figures 7D,E). Rather, ZO-1 immunoreactivity was observed to cytoplasm as compared to sham controls (Figure 7G). To similar extent, endoneurial fluorescence intensity was reduced after CCI, both proximal and distal to the lesion (Figures 7H,I).

### Different Distribution Patterns of ZO-1 and Claudin-1 Immunoreactivity in the Perineurium After CCI

**FIGURE 8:**
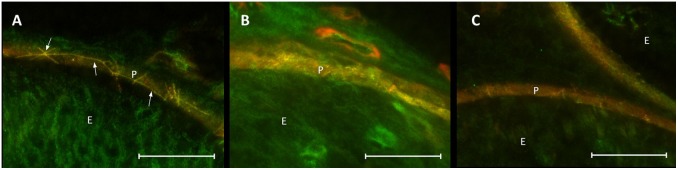
Loss in co-localization of ZO-1 and claudin-1 immunoreactivity after CCI. Compared to sham control **(A)**, merged stainings of ZO-1 and claudin-1 show a decreased overlap of both proteins, both proximal **(B)** and distal **(C)** to the lesion. E, endoneurium; P, perineurium. Arrows point to zig-zag structure of cell-cell contacts in sham controls. Scale bar 50 μm.

Co-staining of ZO-1 and claudin-1 showed a co-localization of immunofluorescent signals for both proteins in the perineurium of sham-operated nerves (Figure 8A). After CCI, the signals were not superimposed: stainings lacked a distinct cell-cell-contact staining in the perineurium or the overlap in sections both proximal and distal to the lesion (Figures 8B,C).

## Discussion

This study shows not only a quantitative decrease in TJP mRNA and immunoreactivity in distinct microstructural compartments but also a disintegration of the perineurial TJP structure in peripheral neuropathy. For analysis of the subcellular distribution in the perineurium, a score/reviewer-based method and a computer-algorithms based method were developed and compared. The latter one matched best with the decrease in claudin-1 mRNA in the whole nerve.

It is known that peripheral neuropathies present with increased permeability of the BNB. In this context, regulation of TJP has been described in several studies. E.g., Moreau et al. (2016) showed a downregulation of claudin-5 and occludin mRNA and occludin immunoreactivity after CCI of the sciatic nerve in rats. This group also demonstrated that claudin-1 mRNA and immunoreactivity was downregulated in CCI of the intraorbital nerve (Moreau et al., 2017). Moreover, Lim et al. (2014) showed a loss of perineurial ZO-1 from cell-cell contacts after partial nerve ligation. In addition to the barrier opening of the BNB, our group observed a decrease of occludin, claudin-1, claudin-5, claudin-19, tricellulin, and ZO-1 in the spinal cord together with an opening of the blood spinal cord barrier (Sauer et al., 2017).

Here, we describe a downregulation of two TJP crucial for the BNB, ZO-1, and claudin-1 in mice after CCI. This parallels the development of the neuropathic phenotype within 7 days. Yet, whole-nerve gene expression analysis is rather unspecific and does not appreciate the complex structure of the nerve, let alone the different compartments of the BNB as outlined above. As Palladino et al. (2017) recently demonstrated via characterization of the normal human BNB transcriptome using laser-assisted microdissection of endoneurial vessels, this part of the BNB is already a hightly complex system with more than 12,000 transcripts including e.g. claudin-5 and ZO-1. Dissecting the anatomical compartments for a more specific approach proves a technical challenge specifically for proteins, due to the need of high amounts of protein for Western blots. Thus, we developed an immunofluorescence-based quantitative approach including the compartmental distribution to specifically semiquantify the proteins. Defining the area of perineurium and endoneurium via the brightfield channel proved reliable (Figure 1). In a following step, these defined areas were placed over the fluorescent images and allowed calculation of average fluorescence intensity in these two areas.

A crucial part of the BNB is the perineurium, embracing one neural fascicle. Its most prominent protein is claudin-1. Its downregulation in the nerve as observed via qPCR could be confirmed and further analyzed using the NIFI method. Notably, images displayed an abundance of claudin-1 in the perineurium as compared to other compartments. This is in line with earlier descriptions (Pummi et al., 2004). Moreover, images of sham-operated nerves showed a distinct perineurial zig-zag pattern of claudin-1 fluorescence indicative of a localization in cell-cell contacts. The observed reduction of claudin-1 immunoreactivity in the perineurium after nerve injury is in line with earlier descriptions (Hirakawa et al., 2003). Interestingly, not only did the signal become less intense after CCI, but also the subcellular distribution changed. In the proximal nerve, no localization in cell-cell-contacts could be identified. In addition, the contrast was much weaker, as confirmed by external blinded reviewers, suggestive of internalization of claudin-1. This reviewer-based score was validated by measuring the number of distinct peaks in fluorescence intensity indicative of cell-cell-contacts per area. The approach also revealed significant differences in perineural peak density between the groups, thus indicating the discrete assembly of claudin-1 in cell-cell-contacts in sham animals and the rather unspecific intracellular distribution in CCI. This method was thus not only an unbiased validation of the external expert opinions but, furthermore, more consistent with the mRNA expression of claudin-1. The observed zig-zag structure reflects the localization at cell-cell contacts; a longer continuous line could represent a long sequence of TJs between cells. A widespread distribution of claudin-1 immunoreactivity in the perineurium, however, suggests a loss of tightness of the barrier. This could lead to an endoneurial edema which in turn could worsen the tightness of the BNB and further loss of TJP in the TJ in a vicious circle (Uceyler et al., 2016). Indeed, removal of TJPs from the TJ and cytoplasmatic internalization of TJP with subsequent barrier breakdown has been described in different circumstances. For example, claudin-1 is internalized in the presence of toll-like receptor 4 (TLR4), leading to diarrhea (Wardill et al., 2016). Occludin is internalized via actin depolymerization (Shen and Turner, 2005). It is thus plausible to postulate an internalization of TJP in the BNB after nerve injury. As TLR-4 is described in peripheral nerve injury, a further investigation into its role in TJP internalization in neuropathic pain would be interesting (Bovin et al., 2007; Koks et al., 2008). Moreover, cytokines, such as vascular endothelial growth factor (VEGF) are known to influence the BNB permeability (Lim et al., 2014). To our knowledge, their effect on TJP internalization has not yet been studied. Strikingly, downregulation was similar in the proximal and the distal part of the ligated nerve confirming a widespread barrier damage throughout the nerve.

ZO-1 is known as a key protein to TJ barriers and widely distributed in tissues. Located in the cytoplasm, it links the TJPs, such as claudins or occludin, to the cytoskeleton. Knockout in MDCK cells caused significant changes in myosin organization at cell-cell contacts and disturbed TJP localization (Tokuda et al., 2014); mice with a homozygous ZO-1 knockdown die (Katsuno et al., 2008). ZO-1 has been described as downregulated and disorganized in partial nerve ligation (Lim et al., 2014). To our knowledge, the present study is the first semiquantification of spatial distribution, and, most importantly, of the accompanying cytoplasmatic redistribution.

Only a TJP and cytoplasmatic anchor protein together can form a functional unit (Poliak et al., 2002). Thus, our observation of a loss in co-localization of claudin-1 and ZO-1 indicates a barrier dysfunction. Indeed, increased permeability after CCI has been observed in earlier studies by our group (Sauer et al., 2017). Quantitative changes *per se* are an important indicator of functional changes, but only a very indirect one. A more specific localization analysis, e.g., for compartments, helps to interpret functional implications. Yet, it is only the high-resolution analysis of localization and structure that promotes a better understanding of changes involved. Thus, the approach of compartment-specific fluorescence-based quantitative and structural analysis allows a deeper understanding of the complex changes involved based on a single method.

In this study, we provide evidence that changes in the BNB after peripheral neuropathy are both quantitative and qualitative. The altered distribution of TJP in the perineurium from cell-cell contacts to the cytoplasm as well as reduced expression of mRNA and protein contribute to the enhanced permeability described earlier (Moreau et al., 2016). As it has been suggested that TJP expression as well as permeability and clinical symptoms might recover or attenuate over time (Hirakawa et al., 2003), it would be interesting to study the further course of perineurial TJP distribution, i.e., whether restructuring of the barrier precedes or follows expression changes. Of similar significance is the order of change (increased quantitative protein/mRNA expression vs. relocalization in the cell-cell-contacts) immediately after damage. Ultimately, further BNB compartments and other TJP, e.g., claudin-5 and ZO-1 in endoneurial vessels, would be an interesting study object.

In summary, we established semiquantitative methods to analyze nerve compartments and cell-cell-contacts using a score and an algorithm for immunofluorescence sections and compared them to mRNA expression. These techniques require only small amounts of tissue sections available from routine nerve biopsies. They could therefore be specifically suitable for patients’ samples when analyzing the BNB and TJP localization in neuropathies of various origins e.g., immune-mediated neuropathies such as GBS or CIDP (Kanda, 2013; Ubogu, 2015). Sera derived from typical patients with CIDP decrease claudin-5 expression and barrier tightness in human peripheral nerve microvascular endothelial cells, prototypical for the BNB. The extent of barrier disruption in the cell line was associated with clinical disability and demyelination in the nerve trunk (Shimizu et al., 2014). In a small study on peripheral nerve biopsies of five patients with demyelinating peripheral neuropathies, claudin-1, and occludin were measured in Western blot and localized by immunolabeling (Manole et al., 2015). Four out of five patients with CIPD had increased claudin-1 protein levels. However, the spatial distribution in nerve sections was not examined. Therefore, quantification methods could be helpful in these diseases to analyze barrier function.

## Author Contributions

AR designed the study, performed the experiments, analyzed the data, and wrote the manuscript. JS performed the experiments and evaluated the data. TL wrote the program for TJ analysis. ES performed the experiments. HR designed the study and wrote the manuscript.

## Conflict of Interest Statement

The authors declare that the research was conducted in the absence of any commercial or financial relationships that could be construed as a potential conflict of interest.
